# Daily intake of non-fried potato does not affect markers of glycaemia and is associated with better diet quality compared with refined grains: a randomised, crossover study in healthy adults

**DOI:** 10.1017/S0007114520000252

**Published:** 2020-01-22

**Authors:** E. A. Johnston, K. S. Petersen, P. M. Kris-Etherton

**Affiliations:** Department of Nutritional Sciences, The Pennsylvania State University, University Park, PA 16802, USA

**Keywords:** Potatoes, Fasting glucose, Cardiometabolic health, Supplemental feeding studies, Diet quality

## Abstract

Epidemiological studies suggest that consumption of potatoes is associated with increased risk of cardiometabolic diseases. However, few clinical trials have empirically tested this. The aim of this single-blind, randomised, crossover study was to evaluate the effect of daily potato consumption, compared with refined grains, on risk factors for cardiometabolic diseases. It was hypothesised that no difference in cardiometabolic endpoints would be detected between conditions, but diet quality would improve with potato consumption. Healthy participants on self-selected diets received one potato-based side dish or one refined grain-based side dish daily, for 4 weeks, separated by a minimum 2-week break. Dishes were isoenergetic, carbohydrate-matched and prepared without excess saturated fat or Na. Participants were instructed to consume the side dish with a meal in place of carbohydrates habitually consumed. Lipids/lipoproteins, markers of glycaemic control, blood pressure, weight and pulse wave velocity were measured at baseline and condition endpoints. Diet quality was calculated, based on 24-h recalls, using the Healthy Eating Index (HEI)-2015. Fifty adults (female *n* 34; age 40 (sd 13) years; BMI 24·5 (sd 3·6) kg/m^2^) completed the present study. No between-condition differences were detected for fasting plasma glucose (–0·05 mmol/l, 95 % CI –0·14, 0·04; *P* = 0·15), the primary outcome or any other outcomes. Compared with refined grains, the HEI-2015 score (3·5, 95 % CI 0·6, 6·4; *P* = 0·01), K (547 mg, 95 % CI 331, 764, *P* < 0·001) and fibre (2·4 g, 95 % CI 0·6, 4·2, *P* = 0·01) were higher following the potato condition. Consuming non-fried potatoes resulted in higher diet quality, K and fibre intake, without adversely affecting cardiometabolic risk.

Suboptimal dietary intake is responsible for approximately 48 % of cardiometabolic deaths (CVD, stroke and diabetes) in the USA^(1)^. Americans typically have poor diet quality and over-consume saturated fat, added sugars and Na, while under-consuming vegetables, which contain dietary fibre and micronutrients including K^(2)^. Potatoes are a rich source of K (about 580 mg/medium potato), a nutrient of public health concern^(2)^. Current consumption in US adults is 2277 mg/d^(2)^, well below the adequate intake level (women 2600 mg/d, men 3400 mg/d)^(3)^. In addition, intake of refined grains exceeds recommendations^(2)^; thus, substitution of refined grains with non-fried/non-boiled potatoes may assist with meeting K recommendations and lower the health burden associated with suboptimal intake of this shortfall nutrient.

In epidemiological studies, higher total potato intake is associated with greater risk of weight gain and diabetes^(4,5)^; neutral associations have been observed for CVD risk^(6)^. A critical limitation of this epidemiological research is the lack of differentiation by the type of potatoes consumed as a function of the cooking method. In observational studies that have examined how differing potato consumption associates with cardiometabolic outcomes^(7)^, French fries/fried potato intake is typically associated with greater risk of weight gain and type 2 diabetes, while non-fried potato intake is less consistently associated with greater cardiometabolic risk^(7)^. These findings suggest that the potato preparation method affects cardiometabolic risk. This evidence has led to uncertainty about whether potatoes should be recommended as part of a healthy diet.

The few randomised, controlled trials that have been conducted have not found adverse cardiometabolic changes with potato consumption^(8–10)^. However, these studies examined potatoes with a high phenolic load that is not representative of potatoes typically consumed by Americans, lacked a comparator condition that reflects real-world dietary substitutions and included relatively small sample sizes. The lack of agreement between epidemiological and interventional research suggests that the preparation method, timing of consumption and background diet may be important modulators of the cardiometabolic effects observed with potato consumption.

In the US diet, potatoes are consumed in a way similar to and interchangeably with refined grains including breads, pasta, rice, orzo and couscous and intake of these foods is greater than that recommended by the 2015–2020 Dietary Guidelines for Americans^(11)^. Therefore, the aim of the present study was to determine the effect of daily potato consumption (one serving/d), compared with an isoenergetic amount of refined grains, on fasting glucose levels, insulin sensitivity, blood pressure (BP), lipids and lipoproteins, arterial stiffness, body weight and dietary intake in healthy males and females. It was hypothesised that after 4 weeks, there would be no difference in fasting glucose levels, insulin sensitivity, BP, lipids and lipoproteins, arterial stiffness and body weight in healthy individuals consuming one potato per d, compared with an isoenergetic amount of refined grains. Moreover, daily consumption of potatoes may improve diet quality.

## Experimental methods

### Study design

**Fig. 1.**
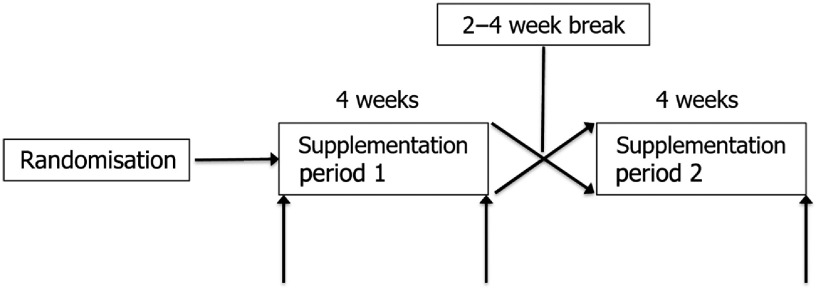
Study design. Up arrows signify testing at baseline (days 1 and 2) and at the end of supplementation periods 1 and 2 (days 28 and 29 of each period).

This was a single-blind, randomised, crossover clinical trial that consisted of two, 4-week intervention periods separated by a 2 to 4-week break (Fig. 1). The primary outcome was fasting plasma glucose; secondary outcomes were weight, insulin, homeostasis model assessment of insulin resistance (HOMA-IR), fructosamine, lipids and lipoproteins, high-sensitivity C-reactive protein (HS-CRP), BP, pulse wave velocity (PWV) and diet quality. Recruitment was completed through StudyFinder and ClinicalTrials.gov. Flyers were also posted in local businesses, offices and around the Penn State campus. Additionally, the study was listed on the webpage of Penn State’s Cardiometabolic Nutrition lab. Data were collected from February 2018 to March 2019.

The present study was conducted according to the Declaration of Helsinki, and all procedures involving human participants were approved by the Penn State Institutional Review Board (STUDY00007854). Participants provided written informed consent at screening prior to enrollment. This trial was registered at clinicalTrials.gov (identifier: NCT03495284).

### Participants

Generally healthy, non-smoking males and females (aged 25–75 years) with a BMI between 20 and 40 kg/m^2^ were recruited for the present study. Exclusion criteria were history of CVD, kidney disease, type 1 or type 2 diabetes (or fasting glucose levels > 7·0 mmol/l), liver disease, cancer or inflammatory conditions (e.g. gastrointestinal disorders, rheumatoid arthritis). Individuals who had lost >10 % of their body weight over the previous 6 months, those with an allergy or intolerance to the study food or any ingredients, women who were pregnant or breast-feeding or had been within the previous year, or any individual taking medications/supplements for elevated lipids, BP or glucose were also ineligible. Individuals consuming >14 alcoholic beverages per week or those not willing to abstain from alcohol consumption for 48 h prior to each test visit were also excluded. Individuals with thyroid disease were eligible if they were on a stable dose of medication for at least 6 months.

Participants completed a telephone screening to assess eligibility. Individuals meeting the inclusion criteria were scheduled for a clinical screening at the Clinical Research Center on the Penn State campus. Participants attended the screening appointment after a 12-h fast and avoidance of alcohol and over-the-counter medication for 48 h. At the screening appointment, height, weight and BP were measured and a fasting blood sample was drawn by trained research nurses. Women of childbearing potential were asked to provide a urine sample to test for pregnancy. Blood samples were sent to a commercial laboratory (Quest Diagnostics) for screening assays (biochemical analysis, complete blood count, lipid profile and fasting glucose).

### Study dishes

The present study consisted of two diet periods, each with a 7-d side dish menu. All dishes were prepared by the Metabolic Diet Study Center at Penn State. Recipes were created by Penn State Cardiometabolic Lab Investigators and the Metabolic Diet Study Center staff (online Supplementary Tables S1 and S2). Participants received six frozen dishes and one refrigerated dish per week. The dishes were commonly consumed potato-based (POT) and grain-based (REF) dishes, made without the addition of excess saturated fat, sugar or Na and contained an average of 837 kJ per serving across diets (Table 1). The potatoes were either steamed or baked (with skin); no side dishes were prepared by frying or boiling. Boiling was avoided to preserve the K content because boiling causes K leeching. A combination of different potato types was used to approximate average American consumption (red, white and gold). Additional ingredients were used to increase the palatability of side dishes (e.g. scallions, onions, breadcrumbs and cheese), but these were kept to a minimum. Participants were told to incorporate the side dishes into a main meal of the day (i.e. breakfast, lunch or dinner) in place of their usual starchy side dish. The dish could be split across two main meals, but participants were instructed to consume the dish in its entirety each day, and not to consume it between meals or as a snack. Participants were also instructed not to consume any potatoes during the refined grain condition and to consume only the potatoes provided during the potato condition.

**Table 1.** Average energy and nutrient composition of the study dishes*

	Energy (kJ)	Fat (g)	SFA (g)	Carbohydrates (g)	Fibre (g)	Protein (g)	Na (mg)	K (mg)
Potato dishes	837	3·7	0·5	37	3·6	6·2	228	826
Refined grain dishes	837	4·1	0·7	34	1·5	6·0	236	119

* Presented mean nutrient values were determined using Food Processor® (ESHA).

Participants completed a 7-d checklist to assess compliance. For each study day, participants indicated whether they consumed their entire study dish and what meal it was consumed with. In addition, any reasons for non-consumption or partial consumption were reported. Compliance checklists and empty containers were returned to the Metabolic Diet Study Center weekly and checked by the centre manager. Compliance was calculated by dividing the total number of days the subject reported consuming the study dish by the number of days in the diet period. Per protocol compliance was assessed as the total number of days the study dish was eaten with a meal, as instructed, by the total number of days in the diet period.

### Study visits

Participants underwent a total of six test visits, two consecutive days at baseline (days 1 and 2) and the end of each diet period (days 28 and 29). Participants were instructed to fast and avoid strenuous physical activity for 12 h and avoid alcohol and over-the-counter medications for 48 h prior to each test visit. Weight was measured using a calibrated electronic scale at each visit; participants wore light clothing and no shoes. Pulse wave analysis and PWV were measured once at each time point. A fasting blood sample was drawn on each test day by trained research nurses.

### Specimen collection and assay methods

Blood samples were collected in a serum separator vacutainer and a sodium fluoride/potassium oxalate vacutainer at baseline (days 1 and 2) and days 28 and 29 of each diet period. Serum was allowed to clot for approximately 30 min at room temperature and then centrifuged for 15 min. Plasma was centrifuged immediately. All samples were frozen at –80°C and analysed in one batch at the completion of the study. Serum was assayed for insulin, fructosamine, lipids and lipoproteins, and HS-CRP at a commercial laboratory (Quest Diagnostics). Plasma was analysed for glucose (Quest Diagnostics). HOMA-IR was calculated using the method described by Matthews *et al.*^(12)^.

### Vascular testing

On 1 day of testing at baseline and the end of each diet period, measures of BP and arterial stiffness, including pulse wave analysis and PWV, were taken using a SphygmoCor XCEL (AtCor Medical) according to the manufacturers’ instructions. After a 5-min rest, trained study staff placed an appropriately sized BP cuff on the participant’s left arm for pulse wave analysis assessment. This test was repeated three times with a 1-min rest between tests. PWV was assessed in the supine position. A BP cuff was placed on the participants’ upper right leg for detection of the femoral waveform. After placement of the cuff, measurements of the linear distance were imputed for estimation of the carotid–femoral distance, a tonometer was placed on the right carotid artery and a 10-s recording of carotid and femoral waveforms were captured; three measurements were taken. For analysis, the last two pulse wave analysis and PWV measurements were averaged.

### Diet quality

Dietary intake was measured by 24-h recalls, administered using the Automated Self-Administered 24-h Dietary Assessment Tool (National Cancer Institute, version 2016), as recommended by the National Cancer Institute Dietary Assessment Primer^(13)^. Participants completed one 24-h recall at baseline, and the midpoint and end of each diet period, for a total of five recalls. Dietary data were reviewed and cleaned as necessary to ensure the study dishes were reported properly. The Healthy Eating Index (HEI)-2015 was calculated using the code developed by the National Cancer Institute^(14)^. Briefly the HEI-2015 consists of thirteen components including four ‘moderation components’ (refined grains, Na, added sugars, saturated fats). Moderation components are reverse scored (i.e. a higher score corresponds to lower intake). Recalls where energy intake was < 2092 kJ were excluded from analysis.

### Statistical analysis

Statistical analyses were performed using SAS 9.4 (SAS Institute Inc.). The data were tested for normality, and transformations were made where necessary; variables transformed for analysis were back-transformed and are presented as geometric means (95 % CI). Mixed-effect models were used to determine endpoint-to-endpoint mean difference and between-condition change from baseline for all outcomes. Condition was included as a fixed effect; subject was included as a random effect. Visit and sex were included as fixed effects to assess for carryover effects and sex differences, respectively. No evidence of order effects or sex differences were detected, so these variables were not included in the final model. For the dietary data, recall time (midpoint or end) was also included as a fixed effect to determine whether differences existed by recall timing; no differences were detected so this was removed from the final model. One recall was excluded due to reported intake < 2092 kJ/d. A power calculation showed fifty participants were required to provide 80 % power (*P* < 0·05) to detect a change in fasting glucose of 0·4 (sd 1·10) mmol/l.

## Results

**Fig. 2.**
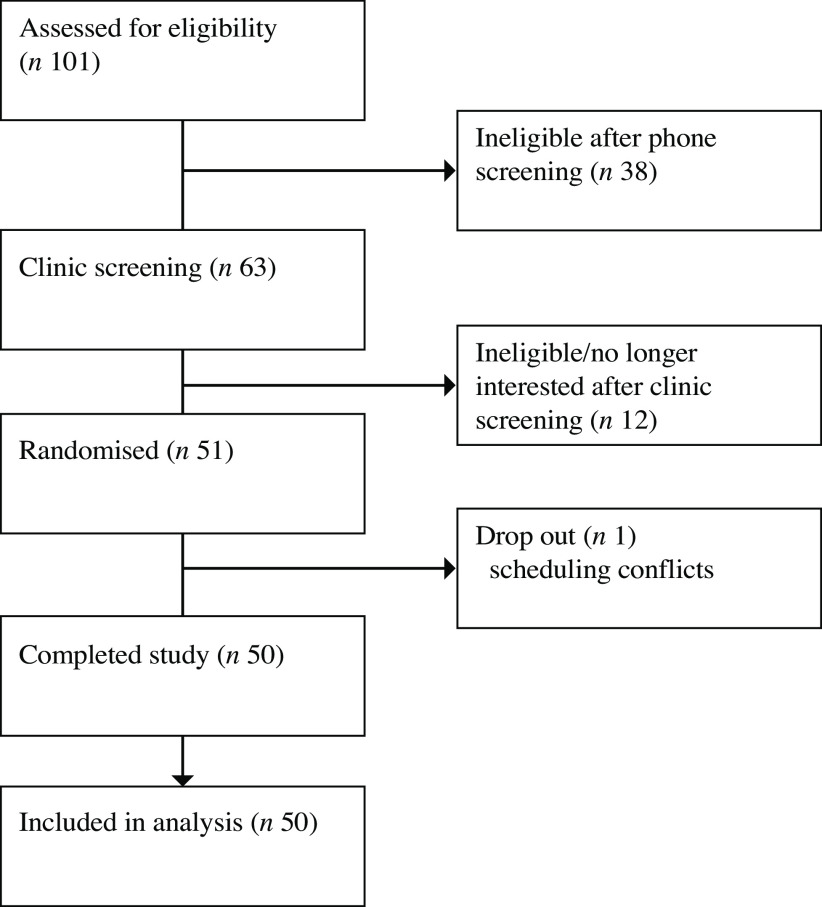
CONSORT diagram.

Fifty adults (female *n* 34) aged 40 (sd 13) years with a BMI of 24·5 (sd 3·6) kg/m^2^ completed the present study. On average, participants reported consuming their study dish on 98 % of study days. In addition, participants consumed the study dish with a main meal on 95 % of study days; consumption with a main meal was slightly higher during the potato condition (95·4 % compared with 93·9 %). One participant dropped out of the study after baseline testing due to scheduling conflicts. All fifty participants who completed the study were included in the analyses (Fig. 2).

### Endpoint-to-endpoint means comparison

No between-condition differences in fasting plasma glucose (POT *v*. REF mean difference –0·05 mmol/l; 95 % CI –0·14, 0·04; *P* = 0·15), insulin (1·47 pmol/l; 95 % CI –3·38, 6·32; *P* = 0·51), fructosamine (–0·09 μmol/l; 95 % CI –2·50, 2·30; *P* = 0·94), HOMA-IR (0·08; 95 % CI –0·09, 0·25; *P* = 0·34) or weight (0·26 kg; 95 % CI –0·12, 0·64, *P* = 0·13) were detected. There were no significant differences in lipids and lipoproteins (total cholesterol –0·04 mmol/l, 95 % CI –0·17, 0·08, *P* = 0·50; LDL-cholesterol –0·04 mmol/l, 95 % CI –0·15, 0·06, *P* = 0·51; HDL-cholesterol –0·02 mmol/l, 95 % CI –0·06, 0·03, *P* = 0·43; TAG 0·96 mmol/l, 95 % CI 0·89, 1·04, *P* = 0·23); HS-CRP (–1·31 nmol/l, 95 % CI –5·66, 3·03, *P* = 0·55) or PWV (0·1 m/s, 95 % CI –0·1, 0·3, *P* = 0·19) following the potato condition compared with the refined grain condition. No between-condition differences were observed in any other measures of BP or arterial stiffness (Table 2).

**Table 2.** Between-condition endpoint-to-endpoint comparison of biochemical and vascular measurements (Least square mean values and 95 % confidence intervals)

	Baseline		Potato		Refined grain		Mean difference		
	Mean	95 % CI	Mean	95 % CI	Mean	95 % CI	Mean	95 % CI	P*
Weight (kg)†	68·1	64·5, 71·9	68·4	64·7, 72·3	68·2	64·5, 72·1	0·26	–0·12, 0·64	0·13
Glucose (mmol/l)	5·3	5·2, 5·4	5·3	5·2, 5·4	5·3	5·2, 5·5	–0·05	–0·14, 0·04	0·15
Insulin (pmol/l)	35·6	29·4, 41·7	37·1	31·0, 43·3	35·6	29·5, 41·8	1·47	–3·38, 6·32	0·51
HOMA-IR	1·2	1·0, 1·5	1·3	1, 1·5	1·2	0·96, 1·4	0·08	–0·01, 2·50	0·34
Fructosamine (μmol/l)	242	236, 247	241	236, 246	241	236, 246	–0·1	–2·5, 2·3	0·94
Total cholesterol (mmol/l)	4·6	4·4, 4·8	4·6	4·4, 4·8	4·7	4·4, 4·9	–0·04	–0·17, 0·08	0·50
LDL-cholesterol (mmol/l)	2·7	2·5, 2·9	2·7	2·5, 2·9	2·7	2·5, 2·9	–0·04	–0·15, 0·06	0·51
HDL-cholesterol (mmol/l)	1·6	1·5, 1·6	1·5	1·5, 1·6	1·6	1·5, 1·6	–0·02	–0·06, 0·03	0·43
TAG (mmol/l)†	0·8	0·7, 0·9	0·9	0·8, 1·0	0·9	0·8, 1·0	0·96	0·89, 1·04	0·23
HS-CRP (nmol/l)	13·7	9·0, 18·4	14·0	9·2, 18·7	15·3	10·5, 20·0	–1·31	–5·66, 3·03	0·55
Vascular measures									
bSBP (mmHg)	116	112, 119	117	113, 120	116	113, 120	0·2	–1·7, 2·1	0·81
bDBP (mmHg)	74	72, 77	74	72, 75	74	72, 75	0·2	–1·4, 1·8	0·83
cSBP (mmHg)	105	102, 108	105	104, 107	105	104, 107	0·1	–1·6, 1·7	0·92
cDBP (mmHg)	75	73, 78	77	72, 77	74	72, 77	0·41	–1·2, 2	0·60
cPP (mmHg)	30	28, 31	31	29, 32	31	29, 33	–0·2	–1·6, 1·1	0·72
AP (mmHg)	5	4, 6	6	4·6, 7·4	6	4·6, 7·4	–0·1	–1·2, 1	0·91
AIx75 %	14	11, 18	15	11, 19	14	10·5, 18	0·9	–1·6, 3·3	0·50
HR (beats/min)	62	59, 65	63	60, 65	62	59, 65	0·7	–1·7, 3·0	0·58
PWV (m/s)	6·6	6·2, 7·0	6·7	6·3, 7·1	6·6	6·2, 7·0	0·1	–0·1, 0·3	0·19
PTT (m/s)	70	67, 73	69	64·6, 73·1	70	66·1, 74·6	–1·5	–7·5, 4·5	0·62

HOMA-IR, homeostasis model assessment of insulin resistance; HS-CRP, high-sensitivity C-reactive protein; bSBP, brachial systolic blood pressure; bDBP, brachial diastolic blood pressure; cSBP, central systolic blood pressure; cDBP, central diastolic blood pressure; cPP, central pulse pressure; AP, arterial pressure; HR, heart rate; PWV, pulse wave velocity; PTT, pulse transit time.

* Endpoint-to-endpoint mean difference.

† Presented as geometric means and 95 % confidence intervals; PROCMIXED (SAS Institute) was used for analyses with participants modelled as a repeated factor.

HEI score during the potato condition was significantly higher than that during the refined grain condition (POT *v*. REF mean difference 3·5, 95 % CI 0·6, 6·4, *P* = 0·01). This was driven by a higher score for both the total vegetable component (0·9, 95 % CI 0·5, 1·3, *P* < 0·001) and the refined grain component (1·5, 95 % CI 0·6, 2·5, *P* = 0·002). No other HEI components differed significantly between conditions (Table 3).

**Table 3.** Between- and within-condition endpoint-to-endpoint mean comparisons and change from baseline for the Healthy Eating Index (HEI) (Least square mean values and 95 % confidence intervals)

Component	Baseline		Endpoint-to-endpoint							Change from baseline					
			POT		REF		Mean difference			POT		REF		Mean difference	
	Mean	95 % CI	Mean	95 % CI	Mean	95 % CI	Mean difference	95 % CI	*P*†	Mean	95 % CI	Mean	95 % CI	Mean difference	95 % CI
HEI total score‡	58·4	54·4, 62·3	55·8	52·6, 59·0	52·3	49·1, 55·6	3·5	0·6, 6·4	0·01	–2·6	–6·3, 1·2	–6·3*	–10·1, –2·5	3·79	0·9, 6·7
Adequacy components															
Total vegetables§	3·9	3·5 4·3	4·0	3·7, 4·3	3·1	2·8, 3·5	0·9	0·5, 1·3	<0·001	0·13	–0·4, 0·6	–0·8*	–1·2, –0·3	0·9	0·5, 1·3
Greens and beans§	2·9	2·2, 3·6	2·4	1·9, 2·9	2·0	1·5, 2·5	0·4	–0·2, 1·0	0·22	–0·4	–1·2, 0·3	–0·9*	–1·6, –0·1	0·4	–0·2, 1·0
Total fruit§	2·7	2·2, 3·3	2·5	2·0, 3·0	2·5	2·0, 3·0	–0·04	–0·5, 0·5	0·89	–0·3	–0·9, 0·3	–0·3	–0·9, 0·3	0·0001	–0·5, 0·5
Whole fruit§	2·8	2·2, 3·5	2·3	1·8, 2·9	2·8	2·3, 3·3	–0·46	–1·0, 0·1	0·09	–0·56	–1·15, 0·04	–0·1	–0·7, 0·5	–0·5	–1, 0·1
Whole grains‖	3·3	2·3, 4·3	3·6	2·7, 4·4	3·4	2·5, 4·2	0·2	–0·7, 1·1	0·64	0·4	–0·9, 1·6	0·1	–1·1, 1·4	0·2	–0·7, 1·1
Dairy products‖	5·9	4·9, 7·0	6·0	5·3, 6·7	6·0	5·3, 6·7	–0·02	–0·8, 0·8	0·95	0·15	–0·9, 1·2	0·17	–0·9, 1·2	–0·02	–0·8, 0·8
Total protein foods§	4·1	3·7, 4·5	3·9	3·6, 4·3	3·9	3·6, 4·3	0·004	–0·4, 0·4	0·98	–0·2	–0·7, 0·3	–0·2	–0·7, 0·3	0·01	–0·4, 0·4
Seafood and plant protein§	3·1	2·5, 3·7	2·7	2·2, 3·2	2·9	2·4, 3·4	–0·2	–0·8, 0·4	0·46	–0·4	–1·1, 0·3	–0·2	–0·9, 0·5	–0·2	–0·7, 0·4
Fatty acid ratio‖	5·6	4·6, 6·7	4·7	3·9, 5·5	4·2	3·4, 5·0	0·5	–0·6, 1·6	0·39	–1·0	–2·3, 0·3	–1·5*	–2·8, –0·2	0·5	–0·7, 1·6
Moderation components															
Na‖	3·6	2·5, 4·7	3·6	2·8, 4·4	3·4	2·6, 4·2	0·2	–0·6, 1·1	0·62	0·04	–1·2, 1·3	–0·25	–1·5, 1·0	0·28	–0·6, 1·1
Refined grains‖	6·9	5·9, 7·9	6·5	5·6, 7·3	4·9	4·1, 5·8	1·5	0·6, 2·5	0·002	–0·4	–1·6, 0·8	–2·0*	–3·2, –0·8	1·6	0·7, 2·6
Saturated fat‖	5·5	4·5, 6·5	5·6	4·8, 6·4	5·3	4·5, 6·1	0·3	–0·6, 1·2	0·51	–0·002	–1·1, 1·1	–0·31	–1·4, 0·8	0·31	–0·6, 1·2
Added sugar‖	8·0	7·2, 8·9	8·1	7·5, 8·7	7·9	7·3, 8·5	0·2	–0·3, 0·8	0·43	0·09	–0·7, 0·9	–0·11	–0·9, 0·7	0·19	–0·4, 0·8

POT, potato; REF, refined grain.

* Significant within-condition change from baseline, *P* < 0·05. PROC MIXED (SAS Institute) was used for analyses with participants modelled as a repeated factor.

† Condition main effect for endpoint-to-endpoint comparison.

‡ Maximum total score 100.

§ Maximum score 5.

‖ Maximum score 10.

Compared with the refined grain condition, K intake was significantly greater following potato consumption (POT *v*. REF mean difference 547 mg, 95 % CI 331, 764, *P* < 0·001). Fibre intake was also significantly greater following potato consumption compared with refined grains (POT *v*. REF mean difference 2·4 g, 95 % CI 0·6, 4·2, *P* = 0·01). There were no significant differences in total energy intake (264 kJ, 95 % CI –268, 795, *P* = 0·32), carbohydrate intake (–9 g, 95 % CI –8, 26, *P* = 0·3) or any other calculated macronutrients or micronutrients between conditions (Table 4).

**Table 4.** Between- and within-condition endpoint-to-endpoint mean comparisons and change from baseline for nutrient and food group intake† (Mean values and 95 % confidence intervals)

	Baseline		Endpoint-to-endpoint mean							Change from baseline					
			POT		REF		Mean difference			POT		REF		Mean difference	
	Mean	95 % CI	Mean	95 % CI	Mean	95 % CI	Mean	95 % CI	*P*‡	Mean	95 % CI	Mean	95 % CI	Mean	95 % CI
Energy content (kJ)	7975	7109, 8845	8485	7828, 9138	8222	7560, 8878	264	–268, 795	0·32	473	–393, 1364	197	–695, 1088	276	–259, 816
Carbohydrate (g)	222	194, 249	241	220, 261	232	211, 252	9	–8, 26	0·30	18	–7·2, 43	8·5	–17, 34	9·5	–8, 27
%E carbohydrates	47	42, 49	48	45, 50	48	45, 50	0·02	–2·6, 2·6	0·99	1·3	–1·7, 4·2	1·2	–1·8, 4·2	0·04	–2·6, 2·6
Protein (g)	85	74, 95	82	74, 90	82	74, 90	–0·2	–8·5, 8·1	0·96	–3·0	–13, 7	–2·9	–13, 7	–0·15	–8·5, 8·2
%E protein	19	17, 21	16	15, 18	17	16, 18	–0·75	–2·3, 0·8	0·33	–2·4*	–4·3, –0·2	–1·5	–3·5, 0·6	–0·78	–2·4, 0·8
Total fat (g)	78	68, 89	84	75, 93	80	71, 89	3·9	–4·7, 12·4	0·37	5·4	–7, 18	1·4	–11, 14	4	–4·6, 12·7
%E fat	37	34, 40	37	35, 39	36	34, 37	1·3	–0·8, 3·3	0·23	0·7	–2·0, 3·4	–0·7	–3·3, 2·1	1·29	–0·8, 3·4
Saturated fat (g)	26	21, 30	26	23, 30	27	24, 31	–0·62	–4·1, 2·9	0·72	0·8	–4, 5·6	1·4	–3·4, 6·2	–0·61	–4·2, 3
%E saturated fat	12	11, 13	12	11, 13	12	11, 13	–0·3	–1·4, 0·8	0·54	0·3	–0·9, 1·4	0·6	–0·5, 1·8	–0·3	–1·5, 0·8
Cholesterol (mg)	250	197, 303	264	218, 309	280	234, 326	–17	–70, 36	0·53	15	–43, 73	30	–28, 88	–15	–68, 38
Fibre (g)	21	18, 25	21	19, 23	18	16, 20	2·4	0·6, 4·2	0·01	–0·1	–3·8, 3·5	–2·6	–6·2, 1·1	2·4	0·6, 4·2
K (mg)	2788	2490, 3086	3044	2809, 3279	2497	2260, 2733	547	331, 764	<0·001	251	–48, 549	–315*	–615, –16	566	347, 785
Na (mg)	3279	2865, 3693	3513	3184, 3843	3512	3180, 3843	1·84	–310, 313	0·99	215	–163, 593	225	–155, 604	–9·5	–320, 301
Total vegetables (c-eq)	1·9	1·7, 2·2	2·2	1·9, 2·4	1·4	1·2, 1·7	0·7	0·4, 1·0	<0·001	0·27	–0·04, 0·66	–0·46*	–0·8, –0·07	0·74	0·43, 1·05
Dark green vegetables (c-eq)	0·4	0·3, 0·6	0·4	0·2, 0·5	0·2	0·1, 0·3	0·14	–0·01, 0·29	0·08	–0·05	–0·2, 0·1	–0·20*	–0·41, –0·01	0·15	–0·01, 0·29
Red/orange vegetables (c-eq)	0·5	0·2, 0·48	0·4	0·3, 0·5	0·4	0·3, 0·5	0·001	–0·131, 0·133	0·99	–0·08	–0·2, 0·1	–0·07	–0·2, 0·1	–0·02	–0·2, 0·1
Starchy vegetables (c-eq)	0·35	0·21, 0·48	1·0	0·9, 1·1	0·1	0·04, 0·18	0·91	0·8, 1·0	<0·001	0·67*	0·5, 0·8	–0·25*	–0·4, –0·1	0·9	0·8, 1·0
White potatoes (c-eq)§	0·3	0·2, 0·4	0·95	0·89, 1·0	0·08	0·02, 0·15	0·87	0·77, 0·97	<0·001	0·66*	0·5, 0·8	–0·23*	–0·4, –0·1	0·9	0·77, 0·97
Total grains (oz-eq)	6·1	4·8, 7·4	6·5	5·6, 7·4	7·3	6·4, 8·2	–0·83	–1·69, 0·03	0·06	0·33	–0·8, 1·5	1·2*	0·1, 2·4	–0·9	–1·71, 0·02
Whole grains (oz-eq)	1·1	0·7, 1·5	1·2	0·9, 1·5	1·1	0·7, 1·4	0·1	–0·2, 0·5	0·43	0·13	–0·3, 0·6	–0·01	–0·5, 0·4	0·14	–0·2, 0·5
Refined grains (oz-eq)	5·0	3·9, 6·2	5·3	4·4, 6·1	6·2	5·4, 7·1	–0·96	–1·8, –0·14	0·02	0·2	–0·9, 1·3	1·2*	0·1, 2·4	–1·02	–1·9, –0·2
Total fruit (c-eq)	1·1	0·8, 1·4	1·1	0·8, 1·3	1·1	0·8, 1·4	–0·06	–0·34, 0·23	0·70	–0·04	–0·3, 0·3	–0·02	–0·3, 0·3	–0·02	–0·3, 0·3
Total dairy products (c-eq)	1·7	1·3, 2·0	1·7	1·4, 2·0	1·8	1·5, 2·0	–0·04	–0·35, 0·27	0·81	0·04	–0·3, 0·4	0·07	–0·3, 0·5	–0·03	–0·4, 0·3
Total protein foods (oz-eq)	5·7	4·6, 6·8	5·7	4·7, 6·8	5·7	4·6, 6·7	0·06	–1·1, 1·12	0·92	–0·05	–1·5, 1·3	–0·16	–1·6, 1·2	0·10	–1·0, 1·2
Oils (g)	29	24, 34	29	23, 34	26	20, 31	3·1	–2·5, 8·8	0·27	–0·14	–6·8, 6·6	–3·7	–10·4, 3·1	3·5	–2·2, 9·2

POT, potato; REF, refined grain; %E, percentage energy; c-eq, cup-equivalents; oz-eq, ounce-equivalents; tsp-eq, teaspoon-equivalents.

* Significant within-condition change from baseline *P* < 0·05.

† Data presented as least square mean values and 95 % confidence intervals unless otherwise stated. PROCMIXED (SAS Institute) was used for analyses with participants modelled as a repeated factor.

‡ Condition main effect for endpoint-to-endpoint mean comparison.

§ Includes all potato types used in the present study and any white flesh-potatoes, excludes sweet potatoes.

### Change from baseline

**Fig. 3.**
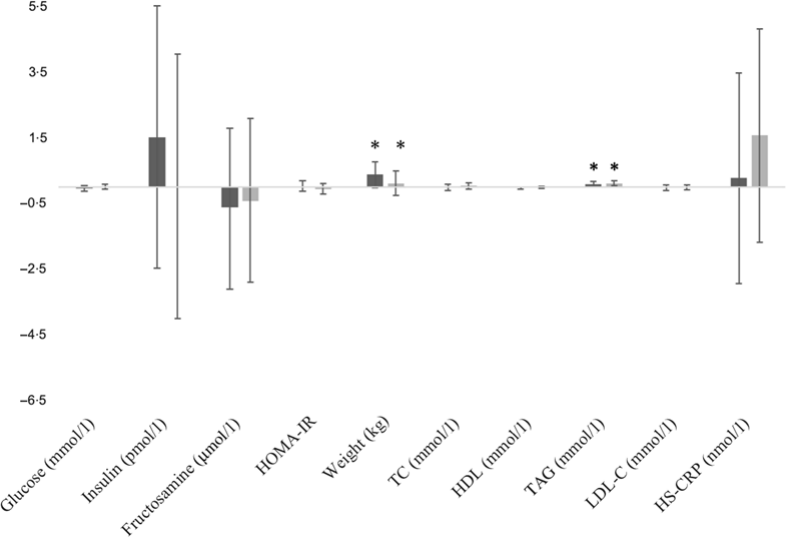
Within- and between-condition change from baseline for all biochemical endpoints. There were no between-condition differences. * Significant within-condition change from baseline (*P* < 0·05); Data are mean values and 95 % confidence intervals; TAG and weight presented as geometric mean values and 95 % confidence intervals. 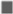, Potato; 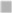, refined grain. HS-CRP, high-sensitivity C-reactive protein.

**Fig. 4.**
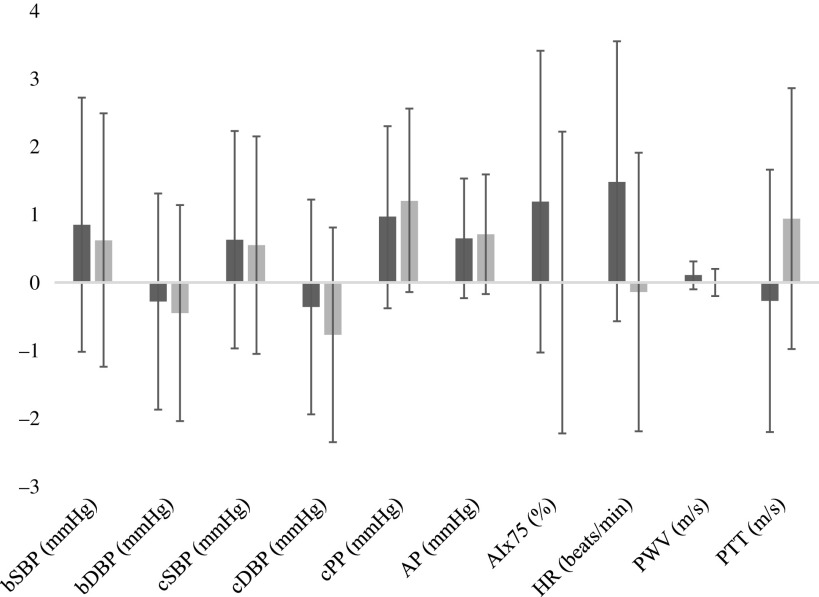
Within- and between-condition change from baseline for all vascular endpoints. There were no between-condition differences. Data are mean values and 95 % confidence intervals. 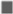, Potato; 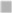, refined grain. bSBP, brachial systolic blood pressure; bDBP, brachial diastolic blood pressure; cSBP, central systolic blood pressure; cDBP, central diastolic blood pressure; cPP, central pulse pressure; AP, arterial pressure; HR, heart rate; PWV, pulse wave velocity; PTT, pulse transit time.

Fasting glucose was unchanged following both conditions (POT *v*. REF –0·04 mmol/l, 95 % CI –0·10, 0·04, *P* = 0·15). TAG significantly increased from baseline following both conditions (POT 0·09 mmol/l, 95 % CI 0·01, 0·17, *P* < 0·001; REF 0·12 mmol/l, 95 % CI 0·04, 0·20, *P* < 0·001), but no between-condition difference was detected (*P* = 0·38). Weight increased slightly from baseline following both conditions (POT 0·38 kg, 95 % CI –0·01, 0·77, *P* < 0·001; REF 0·12 kg, 95 % CI –0·26, 0·50, *P* < 0·001) with no between-condition difference observed (*P* = 0·13). No other between-condition changes from baseline were detected for lipids, lipoproteins, insulin, HOMA-IR, HS-CRP, fructosamine (Fig. 3) or vascular assessments (Fig. 4).

There were no significant changes from baseline in total or component HEI scores following the potato condition. Following the refined grain condition, the HEI total score (–6·3 points, 95 % CI –10·1, –2·5, *P* = 0·002), refined grain HEI component score, (–2·0 points, 95 % CI –3·2, –0·8, *P* = 0·002), total vegetable HEI component score (–0·8 points, 95 % CI –1·2, –0·3, *P* = 0·002) and the greens and beans HEI component score (–0·9, 95 % CI –1·6, –0·1, *P* = 0·03) decreased. The fatty acid ratio decreased on average by 1·5 points from baseline with refined grain intake (95 % CI –2·8, –0·2, *P* = 0·02) (Table 3).

Starchy vegetable and white potato intake increased during the potato condition (0·7, 95 % CI 0·5, 0·8, *P* < 0·001 and 0·7, 95 % CI 0·5, 0·8, *P* < 0·001), and starchy vegetables (–0·2, 95 % CI –0·4, –0·1, *P* = 0·004) and white potatoes (–0·2, 95 % CI –0·4, –0·1, *P* = 0·008) declined with the refined grain condition. Total intake of vegetables and dark green vegetables declined with the refined grain condition (–0·46 oz-eq, 95 % CI –0·8, –0·1, *P* = 0·02 and –0·20 oz-eq, 95 % CI –0·41, –0·01, *P* = 0·04), while total grains (1·2 oz-eq, 95 % CI 0·1, 2·54, *P* = 0·03) and refined grains increased (1·2 oz-eq, 95 % CI 0·1, 2·4, *P* = 0·02) with no change in whole-grain intake in either condition (Table 4).

There was a significant between-condition difference in the change from baseline for K intake (566 mg, 95 % CI 347, 785, *P* < 0·001). Following the potato condition, K intake non-significantly increased from baseline (251 mg, 95 % CI –48, 549, *P* = 0·1) with a significant decrease in K intake following the refined grains condition (–315 mg, 95 % CI –614, –16, *P* = 0·04). Following the potato condition, percentage energy from protein decreased (–2·4 %, 95 % CI –4·3, –0·2, *P* = 0·02) relative to baseline, with no significant change in the refined grain condition (–1·5, 95 % CI –3·5, 0·6, *P* = 0·11) and no between-condition changes from baseline (*P* = 0·33). There were no other significant changes from baseline in any other calculated macronutrients or micronutrients (Table 4).

## Discussion

In this single-blind, randomised, crossover clinical trial, no between-condition differences in fasting glucose, the primary outcome or any other cardiometabolic outcomes were observed, consistent with the hypothesis. In addition, no evidence of adverse clinical effects was observed based on the 95 % CI for glucose, weight, lipids, lipoproteins or vascular outcomes. Furthermore, diet quality measured by the HEI-2015 was greater following the potato condition *v*. the refined grain condition. K and fibre intakes were also higher with potato intake compared with refined grains. The findings of this 4-week study, including healthy participants, suggest there are no adverse cardiometabolic consequences of consuming one medium-sized, unpeeled, baked/steamed potato in place of refined grains such as bread, pasta or rice. Moreover, this dietary modification may increase diet quality, including vegetable intake, and result in greater consumption of fibre and K.

Endpoint-to-endpoint mean comparison showed that fasting glucose was not different between the conditions (*P* = 0·15), and there was no significant change from baseline in either condition or between conditions. Adding further support to this finding is the lack of change in fructosamine, a marker of longer-term glycaemic control. Eligible participants had a fasting glucose level <7·0 mmol/l, although baseline fasting glucose was 5·3 mmol/l, on average, which is approaching the cut point for impaired fasting glucose (>5·6 mmol/l). Different results may have been observed if participants had poorer glycaemic control at baseline; however, a portion of our participants were in the impaired fasting glucose range and we observed no evidence of harm based on the 95 % confidence intervals. Overall, there was no evidence that potato intake increased fasting glucose levels, and although non-significant, the direction of the effect was towards benefit. This is contrary to epidemiological research showing an association between potato intake and risk for diabetes but may be explained by the healthfulness of the study dishes prepared without frying or boiling and without the addition of excess fat or Na. The dishes were also pre-portioned, which limited intake.

We observed no between-condition difference in weight after 4 weeks. We did, however, observe a small increase in weight from baseline following the potato condition (0·38 kg, 95 % CI –0·01, 0·77, *P* < 0·001) and the refined grain condition (0·12 kg, 95 % CI –0·26, 0·50, *P* < 0·001). While there was a small increase in weight from baseline, there was no significant difference between the conditions, which is in contrast to some observational studies^(5)^ that have related potato consumption to weight gain. In support of our findings, Randolph *et al.* found that non-fried potato intake alone did not cause weight gain, rather the preparation method of the potato and how it is incorporated into the diet may explain the weight gain observed in epidemiological research^(9)^. This is consistent with the findings of Erdmann *et al.* who found that when potatoes were eaten at a meal, participants had lower energy intake and had a lower postprandial insulin level than when rice or pasta was eaten^(15)^. This may be explained by the energy density (energy content per g of food) of potatoes. In the present study, the energy density of the potato side dishes was low (2·9–5·0 kJ/g); the refined grain dishes had an energy density in the low and medium range (4·2–12·1 kJ/g). However, in our study, we did not see any difference in energy intake between the conditions, suggesting that energy density of a single dish (resulting in a very small difference in the energy density between conditions) may not explain the lack of weight gain. We expect instead that participants were adherent to the instruction of replacing their usual starchy side dish with the study dish, as instructed, and therefore, there was no significant change in energy intake, which is supported by the dietary data.

We also observed no difference in lipids, lipoproteins, insulin, HOMA-IR, HS-CRP or other biochemical markers between conditions. We observed an increase in TAG from baseline within conditions (POT 0·09 mmol/l, 95 % CI 0·01, 0·17, *P* < 0·001; REF 0·12 mmol/l, 95 % CI 0·04, 0·20, *P* < 0·001), but no between-condition difference was detected (*P* = 0·38). The TAG levels of our participants were within the normal range at baseline (mean 0·82 mmol/l, 95 % CI 0·73, 0·93) and at the end of each period (POT 0·89 mmol/l, 95 % CI 0·79, 1·00; REF 0·92 mmol/l, 95 % CI 0·81, 1·04). It is unclear what caused this change since other lipid/lipoprotein markers did not change and macronutrient intake was not statistically different between conditions. However, there was a non-significant increase in carbohydrate intake from baseline following both conditions, which may have contributed to the increase in TAG (mean difference in change from baseline: 9 g (95 % CI –8, 26), *P* = 0·3). A higher intake of carbohydrates, especially from simple sources and added sugars, is associated with an increase in TAG^(16,17)^, and reductions in carbohydrate intake are associated with improvements in TAG^(18)^.

K has been shown to improve insulin sensitivity, potentially related to its ability to increase insulin secretion^(19)^, which may also explain, in part, why we did not see any adverse effect of potato intake on fasting glucose. K was on average 547 mg higher during the potato condition *v*. the refined grain condition. A double blind randomised controlled pilot study showed that K supplementation, which increased intake by about 2000 mg/d, improved *β*-cell function (about 10 %) and whole-body insulin sensitivity (about 1 %) and reduced insulin resistance (HOMA-IR –0·4)^(20)^. There are several hypotheses related to the mechanism of action, one of which is that K can affect the membrane potential of the *β*-cells of the pancreas, which can increase the release of insulin^(19)^. A meta-analysis of eight prospective cohort studies showed an increase in serum K of about 0·1 mmol/l was associated with a 17 % relative risk reduction in type 2 DM (95 % CI 0·73, 0·95)^(21)^. In the Korean National Health and Nutritional Examination Survey (2008–2010), every one g/d increase in K intake was associated with a 10 % reduction in risk for insulin resistance in female participants with an average intake of <4700 mg/d of K^(19)^. Thus, higher dietary intake of K may lower the risk of insulin resistance and DM, and data from our study suggest that consuming baked/steamed, unpeeled potatoes may assist with increasing K intake.

There was no difference in BP or any measures of vascular health in the present study, despite the increase in K intake. Participants were normotensive at baseline, which may explain this result. The 2017 Guideline for the Prevention, Detection, Evaluation, and Management of High Blood Pressure in Adults cites Class 1, Level A Quality of Evidence for dietary modification to increase K intake for adults with elevated BP or hypertension, unless contraindicated^(22)^. Furthermore, a diet high in K may help to encourage healthy BP regulation, even among individuals following a high-Na diet^(23)^. In a meta-analysis of fifteen randomised controlled trials, increased K intake reduced systolic BP (SBP) by 4·7 mmHg (95 % CI 2·0, 7·0) and diastolic BP by 3·5 mmHg (95 % CI 1·3, 5·7), with a greater reduction in SBP of 6·8 mmHg (95 % CI 4·3, 9·3) and diastolic BP of 4·6 mmHg (95 % CI 1·8, 7·5) in hypertensive participants^(24)^. Some of these studies gave supplements, whereas others used high-K diets as the intervention, with doses from 780 to 4700 mg/d. Based on the meta-regression conducted by Binia *et al.*^(24)^ the difference in K intake we observed between the conditions would be expected to confer a 0·42 mmHg reduction in SBP. Given our sample size, we were underpowered to detect this magnitude of SBP lowering; however, over the long-term, the additional K consumed from a potato may have clinically significant effects on BP control.

Aortic PWV is the ‘gold standard’ for non-invasive measurement of arterial stiffness, a strong predictor of cardiovascular events^(25)^. In the present study, we did not detect any difference in PWV, probably because we included relatively healthy participants and the study was of short duration. However, a 14-d study where 200 g/d of Purple Majesty potatoes was consumed showed a small, but significant change in PWV (–0·3 m/s, *P* = 0·001)^(10)^. No changes in SBP or diastolic BP were observed in that study, and the authors attributed the reduction in PWV to the antioxidants in the purple potatoes.

Overall diet quality was 3·5 points better following the potato condition, compared with the refined grain condition, in the present study. There was no significant change from baseline in overall diet quality in the potato condition, but there was a significant reduction in diet quality following the refined grains condition. The higher diet quality observed during the potato condition relative to the refined grain condition was driven by the increased intake of vegetables (potato) and the decreased intake of refined grains. Based on an analysis of the Nurses’ Health Study I and II and the Health Professionals Follow Up study that found per 10 % increase in diet quality risk of diabetes was reduced by 16 % (HR 0·84, 95 % CI 0·78, 0·90)^(26)^, it is expected that the greater diet quality we observed following the potato condition would confer an approximate 10 % reduction in diabetes risk, if maintained long-term. In an analysis of over 12 400 adults from the Atherosclerosis Risk in Communities Study, participants with the highest, compared with the lowest, HEI-2015 score had a 16 % lower risk of incidence of CVD (HR 0·84, 95 % CI 0·76, 0·93, *P*_trend_ < 0·001), a 32 % lower risk of CVD mortality (HR 0·68, 95 % CI 0·75, 0·89, *P*_trend_ < 0·001) and an 18 % lower risk of all cause-mortality (HR 0·82, 95 % CI 0·75, 0·89, *P*_trend_ < 0·001). These risk reduction estimates persisted even after adjustment for demographic and lifestyle factors, suggesting that improvements in diet quality may reduce the risk of cardiometabolic diseases. Despite better diet quality during the potato condition relative to the refined grain condition, we did not detect any differences in cardiometabolic risk factors in this 4-week study in healthy participants.

The significance of the difference in diet quality observed between the conditions is further demonstrated by the relatively poor diet quality of the participants at baseline. The baseline HEI score (58·4) was below the national average (59)^(27)^. Compared with baseline, diet quality declined with refined grains; no significant difference was detected for the potato condition. At baseline, study participants’ intake of potatoes approximated the national average (0·3 cup equivalents per d)^(28)^. Intake of white potatoes and starchy vegetables increased from baseline during the potato condition and decreased from baseline during the refined grains condition, consistent with study instructions. These findings, combined with the component and food group scores, suggest that when potatoes were added to the diet, they did not displace any core foods but contributed to increasing total vegetable and starchy vegetable consumption. In contrast, when refined grains were given, intake of green vegetables decreased, resulting in a decline in overall diet quality, which contributed to the differences in diet quality between the two conditions (0·14 of the 3·5 point mean difference). The decrease in green vegetable intake during the refined grain condition is unlikely due to other ingredients included in the study recipes (e.g. scallions, celery), as these amounts were very small. It is unclear why green vegetable intake changed, which merits further investigation. Overall, the findings of the present study suggest that replacement of refined grains with potatoes is unlikely to have any adverse dietary effects and may improve alignment with the Dietary Guidelines.

The strengths of the present study include the crossover design and the real-world comparator condition (i.e. refined grains). The study dishes were prepared and portioned in a standard location, so a portion-controlled, consistent quantity was provided. All dishes were prepared without excess saturated fat and Na, and all potato dishes were prepared without boiling or frying, allowing for the study of the impact of healthfully prepared potatoes on cardiometabolic risk factors^(29)^. Dishes were matched for energy and carbohydrates, and compliance was 98 %. In addition, only one enrolled subject did not complete the protocol. The present study also had a number of limitations that should be noted. First, baseline measurements were not taken prior to the second condition; therefore, it is unknown whether subjects returned to baseline following the compliance break; however, order effects were not detected suggesting a return to baseline levels following the break. Compliance was self-reported, which may not reflect actual compliance. Furthermore, dietary data were self-reported, which is prone to recall bias, although this is likely minimised by the repeated within-individual design of the study^(30)^. Additionally, the study had a short duration and included only healthy individuals and therefore is not reflective of long-term dietary change and the results may not be generalised to individuals with disease.

In healthy adults, intake of one medium-sized potato per d for 4 weeks did not change markers of cardiometabolic health compared with one serving of refined grains. Daily consumption of one steamed/baked potato in a healthy portion size prepared without the addition of excess Na or saturated fat can be a part of a healthful diet and may facilitate meeting dietary recommendations.

## Supplementary material

For supplementary material accompanying this paper visit https://doi.org/10.1017/S0007114520000252. click here to view supplementary material
