# Supplementary material for: Daily intake of non-fried potato does not affect markers of glycaemia and is associated with better diet quality compared with refined grains: a randomised, crossover study in healthy adults
Source: Br J Nutr. 2020 Jan 22;123(9):1032–42. doi: 10.1017/S0007114520000252 (PMC7282869; doi:10.1017/S0007114520000252)
Supplement: Supplementary file 1 [file S0007114520000252sup001.docx]

**Supplementary Data**

Supplementary Table 1: Nutrient Profile and Ingredients in the Potato Side Dishes

| Potato Dishes^1^ | Energy (kcal) | Fat (g) | Saturated Fat (g) | Carbohydrates (g) | Fiber (g) | Protein (g) | Sodium (mg) | Potassium (mg) |
| --- | --- | --- | --- | --- | --- | --- | --- | --- |
| Scalloped Potatoes Russet potato (141 g)  White onion (17.7 g)  Margarine (80% fat, 3.9 g)  Flour (4.3 g)  Milk, nonfat (67.8 g)  Salt (0.36 g)  Pepper, ground (0.16 g) | 209 | 3.6 | 0.8 | 39 | 3.5 | 6.7 | 221 | 912 |
| Smashed Potatoes Gold potatoes (140 g)  Garlic, minced (0.8 g)  Greek nonfat yogurt (9.7 g)  Vegetable broth, reduced sodium (9.7 g)  Panko breadcrumbs (18.1 g)  Cheddar cheese, reduced fat (3.2 g)  Scallions (7.7 g)  Sea salt (0.15 g)  Pepper, ground (0.26 g) | 213 | 1.8 | 0.7 | 43 | 3.7 | 7.1 | 231 | 612 |
| Roasted Paprika Potatoes White potato (150 g)  Canola oil (4.1 g)  Paprika (0.61 g)  Sea salt (0.48 g)  Pepper, ground (0.04 g) | 177 | 4.4 | 0.4 | 32 | 3.5 | 3.8 | 200 | 816 |
| Herb Roasted Potatoes Red potato (150 g)  Canola oil (4.4 g)  Thyme (0.11 g)  Rosemary (0.1 g)  Sea salt (0.48 g)  Pepper, ground (0.04 g) | 179 | 4.6 | 0.4 | 32 | 3.3 | 3.8 | 200 | 804 |
| Lemon Potatoes White potato (149 g)  Lemon juice (17.4 g)  Garlic, minced (1.8 g)  Canola Oil (4.2 g)  Parsley (1.8 g)  Sea salt (0.54 g) | 183 | 4.5 | 0.4 | 33 | 3.4 | 4 | 226 | 834 |
| Potato Salad Yellow potato (141 g)  Mayonnaise, fat free (22 g)  Greek yogurt, nonfat (86.6 g)  Celery (17.4 g)  Mustard (5.2 g)  Scallions (13.8 g)  Red onion (9.2 g) | 221 | 1.1 | 0.3 | 42 | 3.5 | 12.3 | 299 | 808 |
| Potato and Spinach Casserole Russet potato (144 g)  Spinach (43.0 g)  Olive oil (5.5 g)  Bouillon cube, reduced sodium (0.9 g)  Panko breadcrumbs (6.5 g)  Sea salt (0.29 g)  Pepper, ground (0.16 g) | 219 | 6.2 | 0.9 | 37 | 4.5 | 5.8 | 219 | 994 |
| AVERAGE | 200 | 3.7 | 0.5 | 37 | 3.6 | 6.2 | 228 | 826 |

^1^ Ingredient weights based on estimated cooked weight

Supplementary Table 2: Nutrient Profile and Ingredients in the Refined Grain Dishes

| Refined Grain Dishes | Energy (kcal) | Fat (g) | Saturated Fat (g) | Carbohydrates (g) | Fiber (g) | Protein (g) | Sodium (mg) | Potassium (mg) |
| --- | --- | --- | --- | --- | --- | --- | --- | --- |
| Garlic Bread Artisan roll (60 g)  Margarine (60% fat, 4 g)  Garlic, minced (2 g) | 193 | 4.6 | 1.2 | 33 | 1.3 | 6.4 | 318 | 25 |
| Couscous Salad Couscous (148 g)  Canola oil (3.7 g)  Fresh dill (0.22 g)  Garlic, minced (0.32 g)  Roasted pepper (14.2 g)  Cilantro (2.96 g)  Salt (0.55 g)  Pepper, ground (0.18 g) | 204 | 4 | 0.3 | 36 | 2.4 | 5.9 | 224 | 130 |
| Spanish Rice Jasmine rice (114 g)  Canola oil (2.1 g)  Bouillon cube, reduced sodium (0.78 g)  Onion (15.4 g)  Poblano pepper (16.2 g)  Canned diced tomato, unsalted (35 g)  Cilantro (0.98 g)  Salt (0.31g) | 194 | 2.5 | 0.3 | 38 | 1.6 | 4.0 | 234 | 297 |
| Red Pepper Rice Jasmine rice (129 g)  Canola oil (3.2 g)  Onion (11.9 g)  Red pepper (15.6 g)  Salt (0.65 g) | 205 | 3.7 | 0.4 | 38 | 0.9 | 3.8 | 253 | 91 |
| Naan Flour (48.2 g)  Sunflower oil (3.6 g)  Greek yogurt, nonfat (10.8 g)  Baking powder (1.2 g)  Salt (0.43 g) | 215 | 4.1 | 0.5 | 38 | 1.3 | 5.6 | 177 | 79 |
| Parmesan Orzo Orzo (104 g)  Canola Oil (3.2 g)  Parmesan cheese, reduced fat (2.65 g)  Salt (0.42 g)  Pepper, ground (0.05 g) | 199 | 4.7 | 0.8 | 32 | 1.9 | 6.6 | 206 | 50 |
| Mac n Cheese Elbow macaroni (65 g)  Cheddar cheese, reduced fat (14 g)  Margarine (60% fat, 5.2 g)  Milk, nonfat (69 g)  Flour (1.9 g)  Salt (0.1 g)  Pepper, ground (0.05 g) | 187 | 4.9 | 1.4 | 25 | 1.2 | 9.9 | 240 | 159 |
| AVERAGE | 200 | 4.1 | 0.7 | 34 | 1.5 | 6 | 236 | 119 |

^1^ Ingredient weights based on estimated cooked weight
